# Artificial intelligence algorithm for predicting cardio-cerebrovascular risk in type 2 diabetes: concordance with clinical and instrumental assessments

**DOI:** 10.1186/s13098-025-01910-6

**Published:** 2025-08-27

**Authors:** Francesco Piarulli, Eugenio Ragazzi, Chiara Celeste Celsan, Annunziata Lapolla, Giovanni Sartore

**Affiliations:** 1 Department of Medicine-DIMED, University of Padova, Padova, Italy; 2 Studium Patavinum, University of Padova, Padova, Italy

**Keywords:** Type 2 diabetes mellitus, Artificial intelligence, Risk evaluation, Cohen’s kappa, Heart disease, Cerebrovascular disease

## Abstract

**Background:**

This study aimed to evaluate the predictive performance of an artificial intelligence (AI)-based algorithm in estimating the risk of cardio-cerebrovascular complications in patients with type 2 diabetes mellitus (T2D).

**Methods:**

Medical records of 532 T2D patients from the Diabetology Unit in Padova, Italy, were analyzed using the Metaclinic AI Prediction Module, which estimates the probability of heart and cerebrovascular organ damage. For patients identified as “Very high” (*n* = 63) or “Low” (*n* = 122) risk for heart disease, additional clinical and instrumental data on their cardiac history were collected. The level of agreement between AI predictions and traditional clinical-instrumental diagnostics was assessed using Cohen’s κ coefficient.

**Results:**

In the “Very high” risk group, the agreement between AI predictions and clinical diagnostics for heart disease was poor (κ = 0.00), while prediction for cerebrovascular disease showed excellent agreement (κ = 0.89). Similarly, in the “Low” risk group, agreement for heart disease remained poor (κ = 0.00), but agreement for cerebrovascular disease was again high (κ = 0.83).

**Conclusions:**

A marked difference was observed in the algorithm’s performance. While the AI showed strong predictive ability for cerebrovascular complications, it failed to reliably predict heart disease risk. These results suggest that the algorithm may be clinically valuable for cerebrovascular risk assessment but needs refinement for cardiac prediction.

**Supplementary Information:**

The online version contains supplementary material available at 10.1186/s13098-025-01910-6.

## Background

Type 2 diabetes mellitus (T2D) is a complex chronic disease characterized by progressive insulin resistance and β-cell dysfunction, leading to sustained hyperglycaemia [1, 2] Its pathogenesis is influenced by both genetic predisposition and modifiable risk factors, such as obesity, poor diet, and physical inactivity. As the disease progresses from a prediabetic state to overt diabetes, persistent hyperglycaemia promotes both microvascular and macrovascular complications through a range of pathophysiological mechanisms including oxidative stress, inflammation, endothelial dysfunction, and atherogenesis [2–4]. Among these mechanisms, glyco-oxidation has emerged as a critical contributor [5, 6]. Recent evidence indicates that in patients with coronary heart disease, glycation and oxidative modifications of human serum albumin display different patterns depending on the presence of T2D, with glycated albumin predominating in subjects with diabetes, while S-thiolated albumin is more common in individuals without diabetes [7].

Among the macrovascular complications, cardiovascular disease (CVD) is the leading cause of morbidity and mortality in individuals with T2D. Patients with diabetes are two to four times more likely to develop coronary artery disease (CAD), with clinical manifestations ranging from stable angina to acute myocardial infarction and sudden cardiac death due to malignant arrhythmias [8]. Also, diabetic autonomic neuropathy can mask the typical symptoms of ischemic heart disease, leading to underrecognition and delayed diagnosis. This highlights the importance of active screening in this high-risk population [9]. The landmark study by Haffner et al. [10] and following studies [11, 12] demonstrated that individuals with T2D and no prior cardiovascular events have a comparable risk of myocardial infarction to patients without diabetes but with established CVD, reinforcing the concept of diabetes as a coronary heart disease equivalent. Consequently, modern guidelines recommend early and aggressive risk stratification and CVD screening in T2D patients, even in the absence of symptoms [13].

Cerebrovascular disease is another major macrovascular complication of diabetes, primarily due to accelerated atherosclerosis affecting both the extracranial carotid arteries and the intracranial vasculature. T2D patients are at increased risk of silent carotid artery stenosis, transient ischemic attacks (TIAs), and both ischemic and haemorrhagic strokes. Early subclinical changes such as increased carotid intima-media thickness and plaque formation are associated with a higher risk of cerebrovascular events [14, 15].

Given that macrovascular involvement may begin even in the prediabetic phase and progress silently, timely identification through targeted screening is crucial. Tools such as the UKPDS risk engine and SCORE2-Diabetes provide tailored risk estimates based on diabetes duration and metabolic control [16, 17]. The 2023 ESC Guidelines recommend classifying most patients with diabetes as having moderate to very high cardiovascular risk, emphasizing the need for aggressive primary and secondary prevention strategies [13].

In this context, improving cardiovascular risk detection and implementing appropriate screening and monitoring protocols are fundamental to reducing the burden of macrovascular complications and improving long-term outcomes in patients with T2D.

The main aim of the present study was to evaluate the ability of an artificial intelligence (AI) algorithm, based on a predefined set of biochemical and anthropometric parameters, to predict the risk of cardio-cerebrovascular complications in patients with T2D. The study compared the algorithm’s predictions with clinical and instrumental data not used by the model, to assess its concordance with traditional diagnostic methods. Ultimately, it sought to determine whether the algorithm can effectively stratify cardiovascular and cerebrovascular risk or requires further refinement, with potential implications for more targeted diagnostic strategies and better risk identification in diabetes care.

## Methods

### Participants

This cross-sectional, single-centre study enrolled a consecutive sample of a total of 532 individuals with T2D comprising 318 males and 214 females, all receiving care at the Unit of Diabetology, AULSS 6 Euganea in Padua (North-East Italy). Participants were eligible for inclusion if they were between 18 and 80 years of age, had a confirmed diagnosis of T2D and were of Caucasian ethnicity. The mean age of the cohort was 71.8 years ± 11.5 SD. Age distribution is illustrated in the bar chart shown in Figure S1. From consecutive patient lists generated during routine departmental visits between October 2023 and June 2024, participants were randomly selected for recruitment. This recruitment strategy enabled the selection of a sample representative of the patient population attending the facility. All participants met the eligibility criteria required for risk prediction analysis using the MetaClinic “AI Prediction Module” [18], a software tool developed for clinical data management in diabetes centres. The study complied with the ethical standards set by the Declaration of Helsinki and subsequent revisions, and received approval from the Ethics Committee of the Province of Padua (study No. 3884/U16/16, protocol No. 0045112, approved on 14.07.2016). Data privacy and processing were managed in accordance with the General Data Protection Regulation (GDPR). All clinical information used for analysis came from existing patient records, including anthropometric and biochemical data. No additional tests beyond routine clinical practice were conducted. Data were anonymized before analysis to ensure confidentiality, and no personally identifiable information was utilized in the AI evaluation. Written informed consent was obtained from all participants prior to inclusion.

### AI system

The MetaClinic platform (developed by METEDA S.r.l., Rome, Italy) is an AI-driven clinical decision support tool designed to estimate the likelihood of developing complications affecting six key organs commonly impacted by diabetes (heart, peripheral arteries, brain, eyes, kidneys, and peripheral nerves) within a two-year period. Integrated into electronic medical records used by Italian diabetes care centres, this tool assists healthcare professionals in the early detection of patients at elevated risk. The algorithmic foundation of the system was originally outlined by Nicolucci et al. [19], employing XGBoost models [20] trained on Electronic Medical Record (EMR) data from the Smart Digital Clinic network (METEDA S.r.l., Rome, Italy), which included longitudinal data from 147,664 individuals managed across 23 diabetes clinics over 15 years. For robustness, external validation was conducted using datasets from five additional diabetes centres, none of which contributed to model training, ensuring the integrity and generalizability of predictions. The validation cohorts ranged from 3,912 to 20,007 individuals [19]. The present analysis applied the AI model to a completely independent cohort. The algorithm targets adults (≥ 18 years) with T2D, provided that at least 40% of a predefined set of 25 clinical and biochemical indicators from the prior year are available. These parameters include anthropometric data (height, weight, body mass index (BMI), waist circumference), vital signs (diastolic and systolic blood pressure), glycaemic markers (fasting blood glucose, postprandial and after breakfast blood glucose, glycated haemoglobin (HbA1c)), renal function indicators (creatinine, estimated glomerular filtration rate (eGFR), microalbuminuria), lipid profiles, liver enzymes, uric acid, creatine phosphokinase, haemoglobin and platelets. While the internal feature importance metrics of the XGBoost model used in MetaClinic platform are not available for this analysis, the input variables were selected based on their established clinical relevance in predicting cardiovascular and cerebrovascular complications in T2D, as described in Nicolucci et al. [19]. The system outputs risk estimates stratified into four categories — low (< 10%), moderate (10–25%), high (25–50%), and very high (> 50%) — based on percentile thresholds (50th, 75th, and 90th) applied to calibrated risk scores over short- (2 years) and intermediate-term (3–5 years) horizons for each organ-specific complication [18].

### Data collection

For each enrolled subject, the digital medical record was processed using the MetaClinic platform’s AI algorithm to predict the risk of developing complications. For the purposes of this study, the focus was primarily on the “heart” module, which assesses the risk of cardiac complications associated with diabetes. The following conditions were considered indicative of pathology: (1) ischemic heart disease: associated with CAD and clinical manifestations such as angina and acute myocardial infarction (AMI); (2) Diabetic cardiomyopathy: functional or structural impairment of the myocardium, initially presenting as diastolic dysfunction (impaired relaxation) and potentially progressing to systolic dysfunction (impaired contractility); (3) Heart failure, defined as New York Heart Association (NYHA) functional class ≥ II; (4) Atrial fibrillation (AF). The algorithm was also able to generate risk predictions in patients with a recorded diagnosis of essential hypertension, without automatically classifying the heart as pathological.

All AI-generated risk scores for cardiac complications were collected and organized in a structured Microsoft Excel (for Microsoft 365 MSO, version 2504 Build 16.0.18730.20122, Microsoft Corporation, Redmont, WA) spreadsheet. Based on the distribution of predicted cardiac risk, the analysis was focused on two subgroups: individuals with a “Very high” risk (*n* = 63; 12% of the sample) and those with a “Low” risk (*n* = 122; 23% of the sample). This stratification allowed for a more meaningful comparative analysis between the two ends of the risk spectrum. For each patient in these subgroups, the digital clinical record was reviewed to extract relevant cardiological history. Specifically, the following were considered: (1) Diagnostic tests: electrocardiogram (ECG), with emphasis on the most recent exam; when available, cardiac echocolor Doppler was also evaluated; (2) Clinical data: cardiology consultation findings and cardiovascular risk factors (e.g., hypertension, dyslipidemia). The ECG served as the principal conventional diagnostic tool for statistical comparison with the AI-generated predictions. Although more advanced modalities such as echocardiography or computed tomography angiography (CTA) offer improved sensitivity for structural cardiac abnormalities, these tools are not routinely available in outpatient diabetes clinics and were not systematically performed across the cohort. ECG, while less sensitive, remains the most widely used and accessible screening tool in this setting and was therefore selected as the primary clinical comparator to the AI model. The following findings in ECG were classified as pathological: Complete right bundle branch block (RBBB); Incomplete or complete left bundle branch block (LBBB); Left anterior fascicular block (LAFB); bradyarrhythmias (atrioventricular blocks, of any type) and tachyarrhythmias (AF, atrial flutter, atrial and ventricular tachycardias); Negative T waves in precordial leads. The following findings were not classified as pathological: Nonspecific ventricular repolarization abnormalities; Sporadic premature ventricular contractions (PVCs); Mild right intraventricular conduction delay.

To provide a more comprehensive evaluation of cardio-cerebrovascular risk, the analysis was expanded to include the “cerebral vessels” module of MetaClinic platform, which estimates the risk of developing cerebrovascular complications, such as TIA and ischemic or haemorrhagic stroke, primarily due to vascular damage. To maintain consistency in the comparative statistical analysis, this extension was applied only to the previously defined subgroups (“Very high” and “Low” cardiac risk). Cerebrovascular risk predictions and diagnostic classifications generated by the algorithm were incorporated into the existing spreadsheet. The distribution of patients according to cerebrovascular risk is shown in Figure S2. As with the cardiac analysis, digital clinical records for each patient in the two subgroups were reviewed for relevant cerebrovascular history. The following elements were considered: (1) Diagnostic tests: color Doppler ultrasound of the supra-aortic trunks (SATs), with priority given to the most recent examination; (2) Clinical data: consultation reports including cerebrovascular assessment and cardiovascular risk factors (e.g., hypertension, dyslipidemia). The SATs measurement result was selected as the principal conventional diagnostic test for comparison with AI predictions. Carotid artery stenosis > 60% was considered as pathological. Intimal thickening or vascular tortuosity were not considered pathological.

### Statistical analysis

All statistical analyses were performed using JMP^®^ Pro Version 17 (SAS Institute Inc., Cary, NC, USA), along with JASP (version 0.18.3, JASP Team, 2024) and jamovi (version 2.5, The jamovi project, 2024), the latter two operating within the R statistical computing environment (version 4.3, R Core Team, 2023). Quantitative continuous variables are reported as mean values with standard deviations (SD). To handle missing data, a pairwise deletion approach was applied, allowing maximum retention of available information. Comparisons of continuous variables were made using either Student’s *t*-test or one-way analysis of variance (ANOVA), while categorical data were assessed using Pearson’s chi-squared test. When appropriate, analysis of covariance (ANCOVA) was used to adjust for potential confounders. Multivariate logistic regression was performed to assess the relationship between selected independent variables (age, disease duration, and sex) and the probability of belonging to a risk group, which served as the dependent variable. Statistical significance was defined as a *p*-value less than 0.05. To evaluate concordance between the AI-based risk classification and the reference standard for diagnosing diabetic cardiac or cerebrovascular pathology, agreement metrics were computed. These included the percentage agreement, and Cohen’s kappa (κ) statistic [21], with interpretation of κ values following the guidelines of Landis and Koch [22], where < 0.00 indicates poor agreement, 0.00–0.20 slight, 0.21–0.40 fair, 0.41–0.60 moderate, 0.61–0.80 substantial, and 0.81–1.00 almost perfect agreement.

## Results

### Patients at “very high” risk of heart disease

The study cohort consisted of 63 patients. The average age was 73.21 y (± 8.93 SD), with a minimum of 52 y and a maximum of 92 y (median age: 74 y; mode: 72 y). The sex distribution showed a slight predominance of males, with 36 men and 27 women. Among male subjects, the average age was 71.67 y (± 8.82 SD), with a minimum of 52 y and a maximum of 88 y (median age: 72.5 y; mode: 63 y). Among female subjects, the average age was 75.26 y (± 8.83 SD), with a minimum of 57 y and a maximum of 92 y (median age: 75 y; mode: 76 y). No significant difference was detected between the sexes (*p* = 0.1152).

**Fig. 1:**
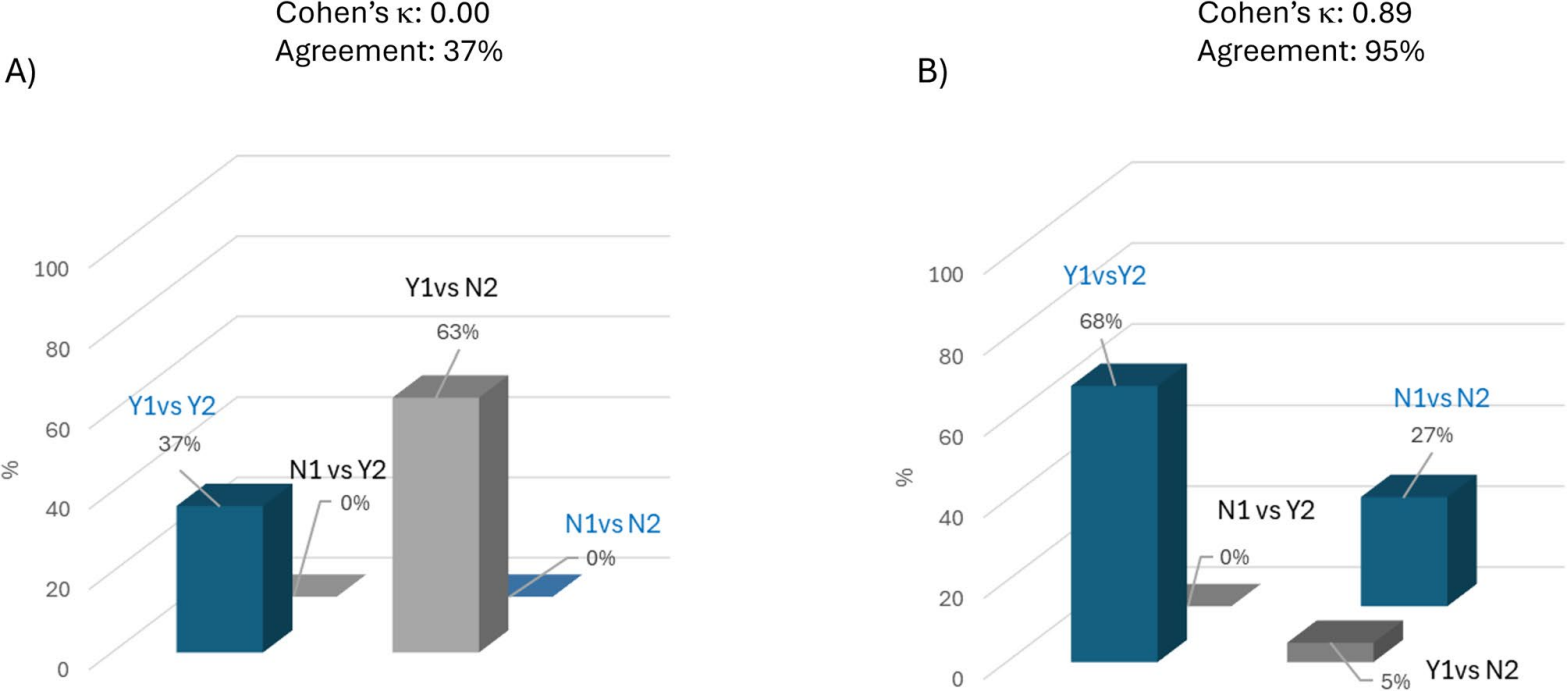
Agreement between the two methods used to evaluate (**A**) heart disease risk and (**B**) cerebrovascular disease risk in patients categorized as “Very High” risk (Method 1: AI software; Method 2: conventional diagnostic tools). Y1 (Yes-1) indicates a high probability of heart disease/cerebrovascular disease risk, and N1 (No-1) indicates a low probability of the risk, according to the AI algorithm. Y2 (Yes-2) and N2 (No-2) indicates the presence/absence of respective risks according to conventional diagnostic tools detailed in Methods section. The percentages displayed above each bar in the chart indicate the distribution of cases across all possible agreement combinations between the two methods. Blue bars denote concordance, while grey bars reflect discordance between the methods

The comparison between the predictions of the AI-based algorithm and the actual clinical and instrumental data regarding the risk of heart disease resulted in Cohen’s κ = 0.00, indicating a poor agreement between the two evaluators (Fig. 1A). Kappa has zero value when two nominal variables are statistically independent [21].

To further explore vascular risk, the analysis was extended (as described in the Methods section) to include cerebrovascular complications by applying the “cerebral vessels” module of the MetaClinic platform to this same cohort. In this context, the agreement between AI-based predictions and traditional clinical diagnostics for cerebrovascular risk was substantially higher, yielding a Cohen’s κ = 0.89 and a 95% percentage agreement, indicating an almost perfect level of agreement (Fig. 1B).

### Patients at “low” risk of heart disease

Initially, the cohort consisted of 122 patients; however, for the purpose of statistical analysis using Cohen’s κ coefficient, only 86 patients were included. The reduction of the sample was necessary for the proper construction of contingency tables, as the digital medical records for 36 patients lacked results from instrumental tests (ECG and/or color Doppler ultrasound of the SATs) making comparison with the algorithm’s predictions impossible.

The mean age was 68.73 y (± 11.94 SD), with a minimum age of 39 y and a maximum of 89 (median age: 70.5 y; mode: 62 y). Overall, the data show greater heterogeneity in terms of age compared to patients classified as “Very high” risk, including the presence of younger individuals. The sex distribution was balanced, with 42 men and 44 women. In male patients, the mean age was 66.48 y (± 12.38 SD), with a minimum of 39 y and a maximum of 88 y (median: 69 y; mode: 71 y). In female patients, the mean age was 70.89 y (± 11.23 SD), ranging from 39 to 89 y (median: 72.5 y; mode: 62 y). No significant difference was detected between the sexes (*p* = 0.087).

**Fig. 2:**
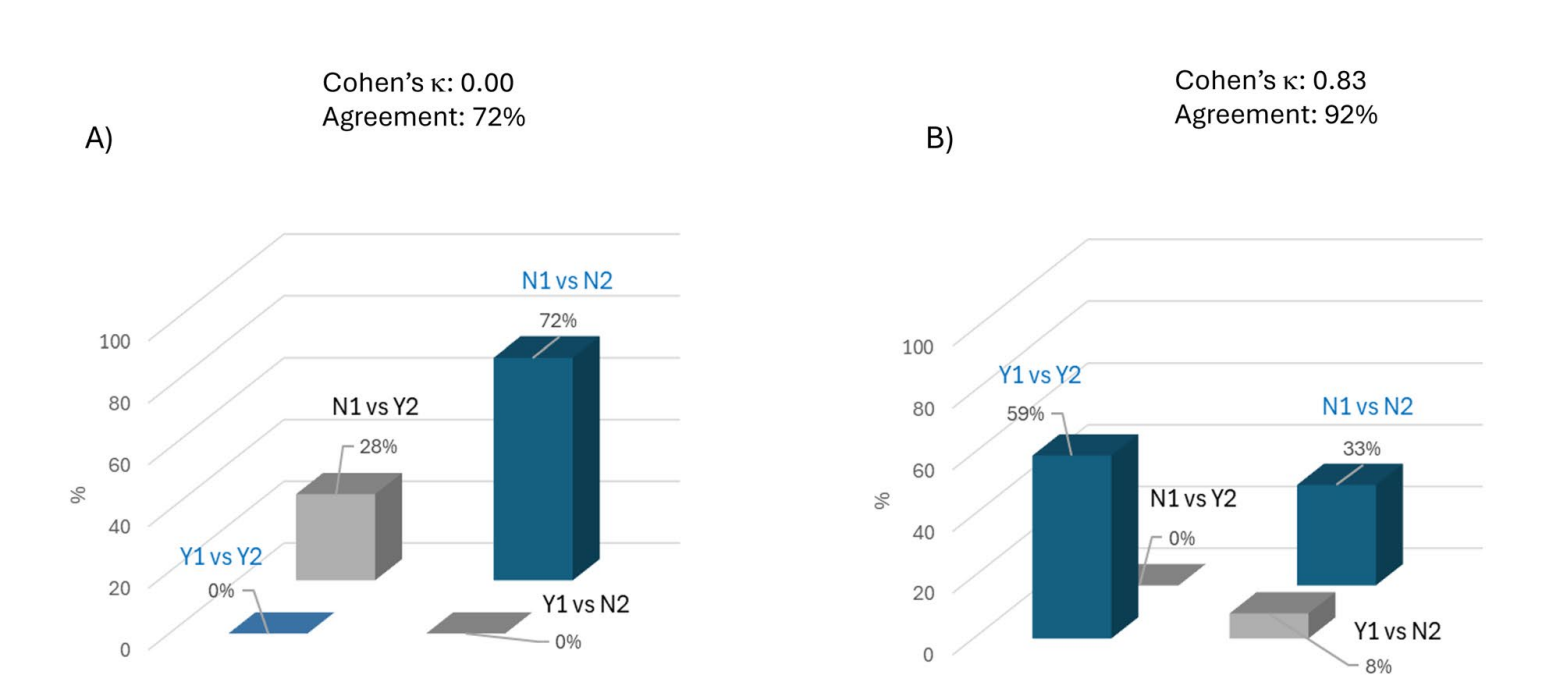
Agreement between the two methods used to evaluate (**A**) heart disease risk and (**B**) cerebrovascular disease risk in patients at “Low” risk (Method 1: AI software; Method 2: conventional diagnostic tools). For explanation of term and abbreviation significance, see Fig. 1

The comparison between the predictions of the AI-based algorithm and the actual clinical and instrumental data regarding the risk of heart disease yielded Cohen’s κ = 0.00 (Fig. 2A), indicating poor agreement between the two evaluators in this group as well—consistent with what was observed in the previously analysed cohort. Despite the percentage agreement was 72%, the zero value of Cohen’s κ is explained by the presence of imbalanced marginal distribution of data.

In contrast, among patients categorized as having low risk of heart disease, the analysis of cerebrovascular risk (performed on the same subgroup using the “cerebral vessels” module) yielded a Cohen’s κ = 0.83. This result again indicates a high level of agreement between the AI-based predictions and traditional clinical diagnostics (Fig. 2B), further supported by a high percentage agreement of 92%.

## Combined analysis of both groups

In light of the results obtained from the separate analyses of the two patient groups, it was considered appropriate to perform a combined analysis of the two cohorts, “Very high” risk together “Low” risk. This decision was prompted by the observation of a significant phenomenon in the contingency tables related to cardiac risk: in both groups, the presence of “zero” values introduced an imbalance in the data, potentially influencing the results. This issue is documented in the literature and is known as the “Cohen’s Kappa Paradox [23].

**Fig. 3:**
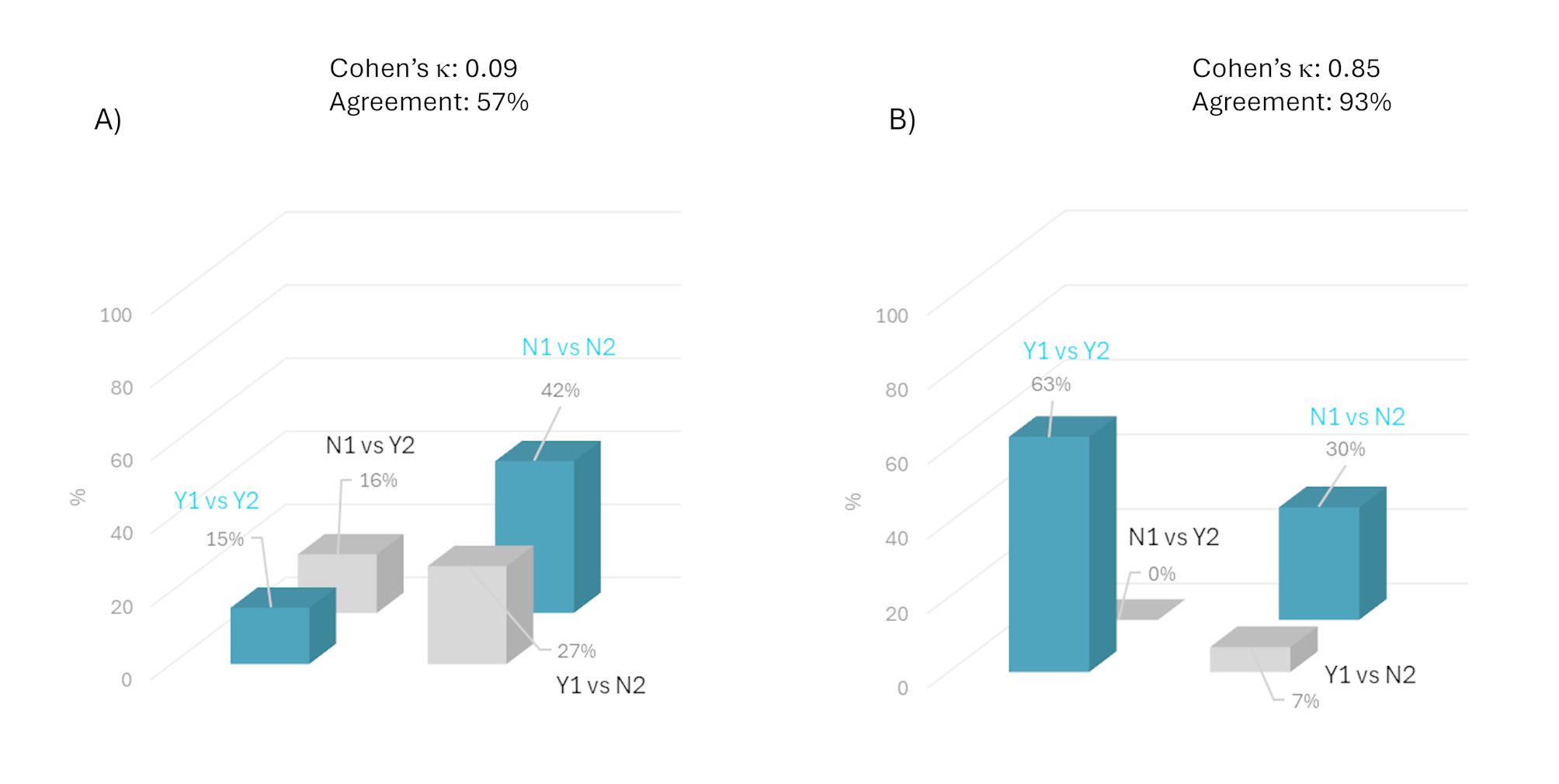
Agreement between the two methods used to evaluate (**A**) heart disease risk and (**B**) cerebrovascular disease risk in combined analysis of the two cohorts, “Very high” risk together “Low” risk patients (Method 1: AI software; Method 2: conventional diagnostic tools). For explanation of term and abbreviation significance, see Fig. 1

The combined analysis yielded a value of Cohen’s κ = 0.09 for cardiac risk. Although this represents a slight improvement compared to the total lack of agreement observed in the two groups when analyzed separately, the level of agreement between the algorithm’s predictions and traditional diagnostic tools remains low (Fig. 3A). On the other hand, the Cohen’s κ for the risk of cerebrovascular disease was 0.85, once again confirming the strong concordance between the algorithm’s predictions and the clinical-instrumental data (Fig. 3B), also supported by the high percentage agreement value of 93%.

### Biochemical and anthropometric parameters considered by the algorithm

The biochemical and anthropometric parameters taken into account by the predictive AI algorithm and available in the digitized medical records are shown in Table 1. Additional parameters not used by the algorithm are also shown. Statistically significant differences between the two cohorts of “Very high” risk and “Low “risk patients were found for the following parameters: age, disease duration, weight, BMI, waist circumference, diastolic and systolic blood pressure, AST, postprandial blood glucose and eGFR (Table 1).

**Table 1:** Anthropometric and biochemical parameters in the two patients’ groups

Parameter	“Low” risk group (*n* = 122)	“Very high” risk group (*n* = 63)	*P* ^†^
^a^Age, y	67.8 ± 12.8	73.2 ± 8.9	**0.003**
^a^Gender, M/F	58/64	36/27	0.2157
Height, cm	166.3 ± 10.5	167.1 ± 9.2	0.5953
Weight, kg	76.3 ± 17.7	84.7 ± 15.6	**0.0017**
BMI, kg/m^2^	27.5 ± 5.3	30.4 ± 5.5	**0.0009**
Waist circumference, cm	97.4 ± 13.9	105.4 ± 10.9	**0.0015**
^a^Disease duration, y	11.8 ± 10.3	16.1 ± 7.8	**0.004**
Diastolic blood pressure. mmHg	77 ± 10	81 ± 9	**0.0151**
Systolic blood pressure, mmHg	137 ± 21	150 ± 21	**< 0.0001**
Platelets, 10^3^/mm^3^	254 ± 66	237 ± 48	0.2368
Microalbuminuria, mg/L	17.9 ± 38.9	30.0 ± 46.0	0.3524
Gamma-glutamyl transferase, IU/L	34.8 ± 65.0	33.4 ± 23.8	0.9394
Haemoglobin, g/dL	13.9 ± 1.7	13.2 ± 2.3	0.1634
Total serum cholesterol, mg/dL	169.7 ± 40.5	159.7 ± 29.3	0.0939
LDL (Friedewald formula), mg/dL	93.5 ± 35.3	82.9 ± 24.8	0.0510
HDL cholesterol, mg/dL	53.5 ± 12.7	51.8 ± 12.3	0.4080
Triglycerides, mg/dL	120.6 ± 75.4	120.3 ± 61.6	0.9795
AST, IU/L	21.6 ± 7.9	25.6 ± 13.5	**0.0457**
ALT, IU/L	22.7 ± 12.9	26.1 ± 16.0	0.2299
Uric acid, mg/dL	5.3 ± 1.4	5.1 ± 0.8	0.8106
Fasting blood glucose, mg/dL	140.7 ± 47.0	149.0 ± 38.4	0.2440
Post-prandial blood glucose, mg/dL	138.1 ± 40.1	215.7 ± 83.5	**0.0013**
Blood glucose after breakfast, mg/dL	149.9 ± 53.9	153.1 ± 47.0	0.7530
HbA1c, % (mmol/mol)	7.2 ± 1.5 (55 ± 16)	7.2 ± 1.1 (55 ± 12)	0.9356
Serum creatinine, mg/dL	0.85 ± 0.33	0.94 ± 0.27	0.0761
eGFR, ml/min/1.73 m^2^	84.3 ± 20.2	75.9 ± 19.0	**0.0108**

Data are expressed as mean ± SD

AST: Serum Aspartate Aminotransferase; ALT: Serum Alanine Aminotransferase

^a^ Parameter not used by AI prediction algorithm

^*†*^ Statistical significance was assessed by Student’s *t-*test for continuous data, and by Pearson’s chi-squared test for categorical data

### Disease duration

Although not considered in the AI algorithm, data regarding disease duration, expressed in years since diagnosis, were evaluated for the two cohorts of patients. Patients considered to be at a “Very high” risk of heart disease (*n* = 63) had an average disease duration of 16.1 ± 7.8 years, while those classified as “Low” risk (*n* = 122) had an average duration of 11.8 ± 10.3 years. The difference between groups was statistically significant (*p* = 0.004). Analysis of covariance confirmed the significance of the risk factor (*p* = 0.003), while ruling out a significant influence of gender (M/F) (*p* = 0.297). Furthermore, no significant interaction was found between risk factor and gender (*p* = 0.448). These results highlight that the duration of disease is longer in patients with higher risk, regardless of gender, suggesting that a longer period of exposure to diabetic pathology may negatively impact cardio-cerebrovascular risk.

A multivariate logistic regression analysis was also conducted to assess the association between variables considered “independent” – age, disease duration, and gender – and the probability of belonging to the “Very high” risk group as opposed to the “Low” risk group. The results showed that only age was a statistically significant indicator of risk (*p* = 0.0352). The other two variables – disease duration and gender – did not reach statistical significance, with *p* = 0.0886 and *p* = 0.0796, respectively. Therefore, an increased probability of falling into the higher risk group emerges as age increases. A subsequent logistic regression multivariate analysis was conducted excluding the variable “age” and considering only disease duration and gender. In this case, disease duration showed a higher level of significance (*p* = 0.0026), confirming its role in increasing risk. The variable gender, however, remained non-significant (*p* = 0.1305).

## Discussion

The application of AI in predicting the risk of complications associated with diabetes is an area of growing interest in clinical practice. The present study investigated the predictive capability of the MetaClinic AI Prediction Module in stratifying the risk of heart disease and cerebrovascular disease, comparing the algorithm-generated predictions with clinical and historical data not included in the algorithm itself, as well as with instrumental diagnostic reports from patients followed at our Diabetology Unit in Italy. Our study fits within the context of existing literature evaluating the use of AI in cardiovascular risk prediction among patients with T2D. In particular, it follows recent findings from our research group, indicating that the use of an optimized AI algorithm can effectively estimate the risk of developing diabetic neuropathy, thereby suggesting more targeted and in-depth screening for at-risk patients [24]. The present study harmonizes with broader trends reported in recent systematic and narrative reviews. Kee et al. [25] identified substantial heterogeneity in prediction targets, input features, and validation methods across machine learning models for cardiovascular complications in diabetes, emphasizing the lack of models that incorporate multimodal clinical and instrumental data. Similarly, Oikonomou and Khera [26] highlighted the shift toward integrating AI with precision diagnostics, including imaging and ECG data, as in the model presented by Raghunath et al. [27]; Wang Y. et al. [28] contextualized in a narrative review their approach within broader trends in machine learning for cardiovascular risk prediction and phenotyping, including in patients with diabetes. Also, Wang M. et al. [29] conducted a systematic review analysing various AI models applied to predicting CVD risk in patients with T2D. They found that the most frequently used predictive factors were age, BMI, blood pressure, and cholesterol levels. These findings align with our study, particularly regarding the significant role of age, which was confirmed by multivariate logistic regression despite not being included among the variables considered by the AI algorithm. Additionally, the role of BMI and blood pressure as relevant biochemical and anthropometric parameters was supported by their inclusion in the algorithm. The review [29] also highlighted limited reproducibility across many models, with only one externally validated among the studies analysed. Our results partially support this finding: while the algorithm demonstrated good stratification capability for cerebrovascular risk (κ = 0.89 in “Very high” risk patients, κ = 0.83 in “Low” risk patients, κ = 0.85 in the merged sample), its ability to predict heart disease risk was scarce (κ = 0.00 in both “Very high” and “Low” risk patients, κ = 0.09 in the merged sample). Some of the AI-based models, proposed for predicting cardiovascular outcomes in patients with T2D or in broader populations, are evaluated using statistical discrimination metrics such as the area under the curve (AUC); conversely, our study focused on real-world agreement with clinical and instrumental assessments, quantified using Cohen’s κ. While not directly comparable, population-based models such as those by Weng et al. [30] and Bergamini et al. [31] have demonstrated AI’s potential in stratifying cardiovascular and cerebrovascular outcomes. However, these models are typically validated using population-level event data and statistical discrimination metrics. Our approach instead emphasizes real-world concordance with instrumental diagnostics such as carotid ultrasound, highlighting MetaClinic’s potential utility in clinical workflows for the early identification of cerebrovascular risk in patients with T2D. While our sample size is relatively small, the strong agreement for cerebrovascular outcomes supports the potential clinical utility, particularly for secondary prevention and targeted screening.

Another contribution to this field is provided by Sang et al. [32], who developed and validated a machine learning-based AI model for CVD prediction in T2D patients. This model was specifically tailored to the Korean population to overcome the limitations of traditional predictive tools. One of the most relevant aspects of this study is its primary outcome – onset of CVD within 3 years. Notably, 10.2% of the discovery cohort experienced CVD, which is especially relevant to our study, since 12% of our patients were classified by the algorithm as “Very high” risk for heart disease. The key risk factors identified by Sang et al. [32] also provide interesting comparisons. Specifically, in their study creatinine levels and HbA1c emerged as the main predictors of cardiovascular risk. A parallel can be drawn with our findings, where the eGFR was statistically significant among the algorithm’s input variables (*p* = 0.0108) to distinguish between the two patients’ groups. Conversely, while Sang et al. [32] identified HbA1c as a key risk factor independently of average blood glucose, in our study, postprandial glucose was statistically significant (*p* = 0.0013). Among the several key factors identified in the study by Sang et al. [32] on the Korean population — namely AST, ALP, ALT, BMI, and antihypertensive therapy (calcium channel blockers and diuretics) — AST (*p* = 0.0457) and BMI (*p* = 0.0009) emerged as statistically significant in our analysis.

A particularly relevant study to our work is that of Nicolucci et al. [19], who developed predictive models for T2D complications using data from an Italian cohort of 147,664 patients followed over 15 years. The study represents the foundational work for the development of the AI algorithm used in our research. It is also especially relevant given that our investigation was conducted on patients attending an Italian centre. Nicolucci et al.’s models [19] achieved an accuracy above 70% for all analysed complications. Validation across five independent centres confirmed the models’ performance, although some variation was observed—particularly for cardiovascular complications, where one centre reported an AUC below 0.60. This is pertinent to our study, in which predictions for cerebrovascular risk aligned well with traditional diagnostic methods, whereas predictions for heart disease risk demonstrated lower accuracy.

Regarding predictive factors, Nicolucci et al. [19] did not identify a single dominant parameter but rather a complex combination of variables, each contributing less than 5%. This contrasts somewhat with our study and that of Sang et al. [32], where some biochemical markers showed clear statistical significance for cardiovascular risk prediction.

The strong agreement found in the present investigation between the AI predictions and traditional diagnostics for cerebrovascular disease underscores the potential clinical utility of the AI model in routine diabetes care. In practice, these predictions could be used to prioritize patients for advanced neuroimaging (e.g., carotid Doppler ultrasound, brain magnetic resonance imaging) and neurology referrals, especially in resource-limited settings where routine screening for subclinical cerebrovascular disease is not feasible for all patients. For patients flagged as “Very high” risk, clinicians could initiate earlier or more aggressive preventive strategies, including tighter blood pressure control, antiplatelet and statin therapy consideration, or lifestyle interventions. Conversely, patients classified as “Low” risk could potentially avoid unnecessary diagnostic procedures, reducing healthcare burden and cost. By integrating the AI tool into electronic health records or decision-support systems, clinicians might be alerted in real time to patients needing cerebrovascular risk reassessment, enhancing preventive care pathways in T2D populations.

Our study has limitations. One of the main limitations concerns the presence of null values in the contingency tables for heart disease risk analysis. This is a documented phenomenon known in the literature as “Cohen’s Kappa Paradox” [23]. It occurs when the Cohen’s κ coefficient is influenced by marginal data distribution, potentially underestimating actual agreement even when high concordance exists. In our study, zero values in the contingency tables may have contributed to the low κ values for heart disease risk, possibly leading to an inaccurate assessment of the algorithm’s predictive ability. Another critical point relates to the delayed extension of the analysis to cerebrovascular risk, which was applied only to patients previously selected based on predicted heart disease risk. This methodology led to a more heterogeneous sample for cerebrovascular risk assessment, since patients were not originally selected based on cerebrovascular risk. Consequently, risk stratification for cerebrovascular complications was based on a group that included all risk levels, not just those classified as “Very high” or “Low” according to the “brain vessels” organ-specific risk sheet of the AI prediction algorithm. A final notable limitation concerns the instrumental diagnostics used for comparison with the algorithm’s predictions. For cerebrovascular risk, the main exam considered was the color Doppler ultrasound of the SATs, a highly accurate test for detecting cerebrovascular abnormalities. For heart disease risk, the main diagnostic tool used in this study was the ECG. While ECG is widely employed as a frontline screening method for detecting cardiac anomalies in patients with diabetes [33], it provides limited structural and functional information [34] compared to more advanced modalities such as echocardiography, which is considered the gold standard for comprehensive cardiac assessment [35], or also CTA; however, both are resource-intensive and not systematically available across centres, especially in large-scale, real-world registries. The reliance on ECG was primarily due to its broader availability in the studied population, as not all patients had access to echocardiographic evaluation; moreover it is inexpensive, and routinely performed in most diabetes care settings. Notably, we observed several cases of apparent discordance between the AI-based risk predictions and ECG findings, particularly within the “Very High” risk group. In these cases, the algorithm flagged individuals as having elevated risk, despite ECG results not showing overt pathological changes. However, this discrepancy should not be interpreted as a shortcoming of the AI model. Rather, it likely highlights the limited sensitivity of ECG in detecting subclinical or early-stage cardiac dysfunction. Indeed, ECG often fails to reveal abnormalities that may be evident only through more sensitive or multimodal assessments. This reinforces the potential of AI algorithms, trained on diverse clinical and laboratory data, to detect latent risk not captured by conventional diagnostics. Thus, rather than contradicting each other, the AI predictions and ECG findings may reflect different dimensions of risk, underscoring the role of algorithmic tools as complementary to, rather than redundant with, traditional screening methods.

Another limitation of the present study is the absence of a direct comparison between our AI-based risk prediction algorithm and established clinical risk assessment tools such as the UKPDS Risk Engine and SCORE2-Diabetes. These tools, while widely used and recommended in recent guidelines [16, 17], rely primarily on demographic and metabolic parameters, and may not fully capture early or subclinical cardiovascular and cerebrovascular changes detectable through instrumental modalities such as ECG. Therefore, their predictive accuracy in certain patient subsets, especially in the preclinical or early diabetic phase, may be limited. Future research from our group will aim to incorporate a direct comparative analysis between AI-based models and these traditional risk engines, with the goal of evaluating whether integration of instrumental data provides incremental value in individualized risk stratification.

Building on the promising predictive performance of the AI model for cerebrovascular complications, a prospective validation study could be considered to assess its clinical utility in real-world settings. Such a study would ideally involve longitudinal follow-up of a new cohort of T2D patients, tracking cerebrovascular outcomes over time to evaluate the model’s predictive accuracy and its potential impact on clinical decision-making. It may also be valuable to examine how integrating the AI tool into routine care pathways, for example, by flagging high-risk individuals during outpatient visits, so influencing diagnostic testing rates, preventive strategies, and patient outcomes. These steps would be important to determine the algorithm’s applicability and effectiveness in everyday clinical practice.

## Conclusions

Despite the aforementioned limitations, this study offers valuable insights into the use of AI for predicting cardio-cerebrovascular risk in patients with T2D. However, to strengthen the validity of the observed results, future studies with larger patient cohorts and more homogeneously selected risk groups will be necessary. Additionally, follow-up of patients classified as “Very high” risk is warranted to monitor the potential onset of major cardiovascular events.

## Supplementary Information

Below is the link to the electronic supplementary material.

Supplementary Material 1

## Data Availability

The datasets generated and/or analyzed in the current study are available from the corresponding author upon reasonable request.

## Abbreviations

AF: Atrial fibrillation
AI: Artificial intelligence
ALP: Alkaline phosphatase
ALT: Serum Alanine Aminotransferase
AMI: Acute myocardial infarction
ANCOVA: Analysis of covariance
ANOVA: One-way analysis of variance
AST: Serum Aspartate Aminotransferase
AUC: Area under the curve
BMI: Body mass index
CAD: Coronary artery disease
CTA: Computed tomography angiography
CVD: Cardiovascular disease
ECG: Electrocardiogram
eGFR: Estimated glomerular filtration rate
EMR: Electronic medical record
GDPR: General data protection regulation
HbA1c: Glycated haemoglobin
HDL: High-density lipoprotein
LAFB: Left anterior fascicular block
LBBB: Left bundle branch block
LDL: Low-density lipoprotein
NYHA: New York Heart Association
PVCs: Sporadic premature ventricular contractions
RBBB: Complete right bundle branch block
SATs: Supra-aortic trunks
SD: Standard deviation
T2D: Type 2 diabetes mellitus
TIA: Transient ischemic attack
