# Supplementary material for: Artificial intelligence algorithm for predicting cardio-cerebrovascular risk in type 2 diabetes: concordance with clinical and instrumental assessments
Source: Diabetol Metab Syndr. 2025 Aug 27;17:361. doi: 10.1186/s13098-025-01910-6 (PMC12382123; doi:10.1186/s13098-025-01910-6)
Supplement: Supplementary file 1 — Supplementary Material 1 [file 13098_2025_1910_MOESM1_ESM.pdf]

Supplementary information

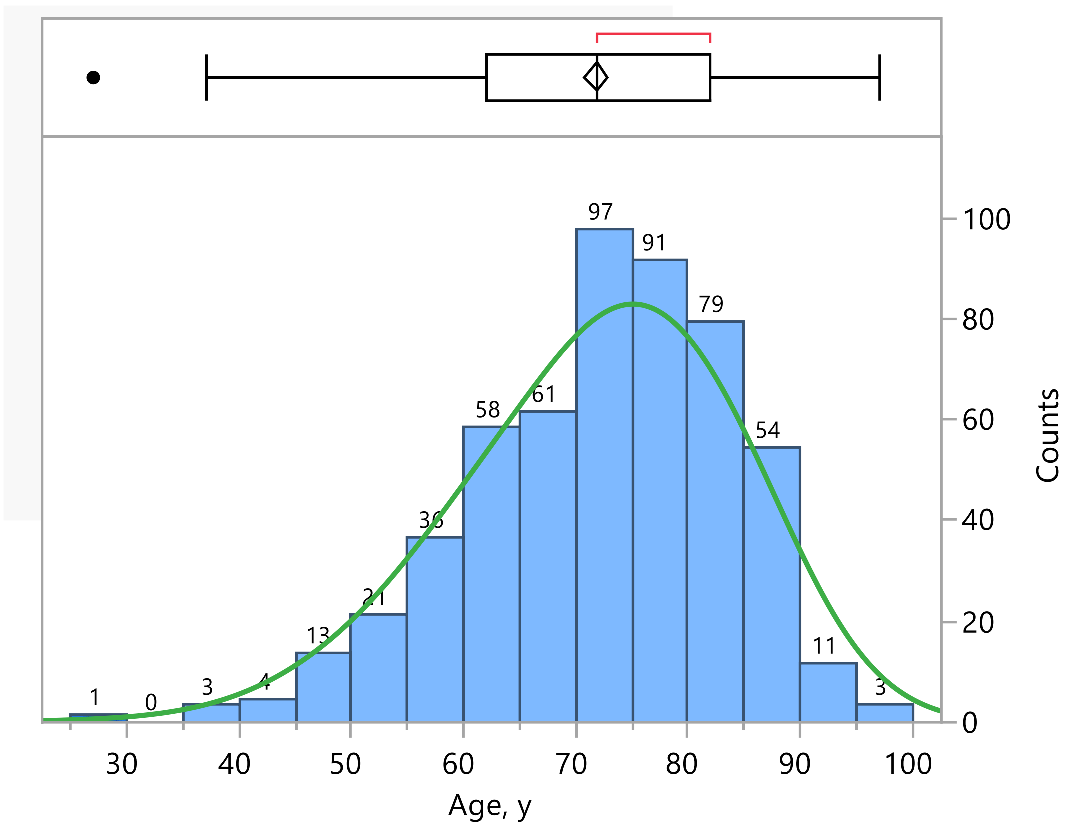

Fig. S1. Age distribution of the entire patient sample (*n* = 532). The number of subjects in each age interval is shown above the corresponding bar. The superimposed curve represents the theoretical continuous interpolation of the values.

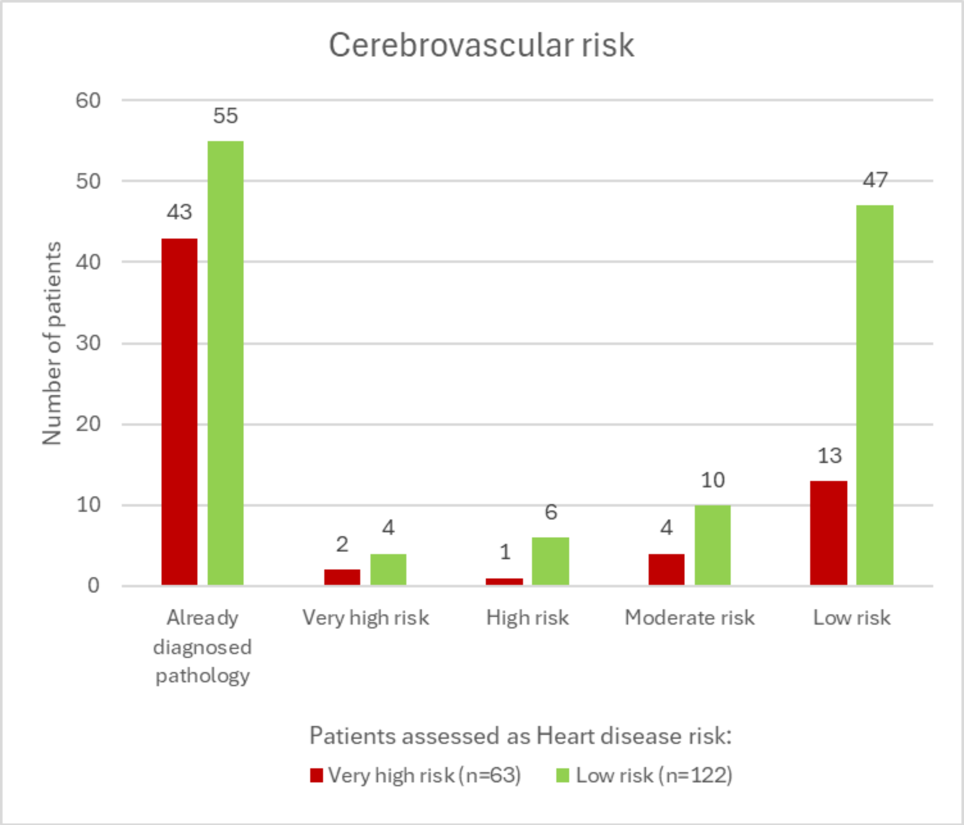

Fig. S2. Distribution of cerebrovascular risk, according to AI algorithm, in the two groups of patients already identified for heart disease risk “Very high” and “Low”, respectively.
